# Neuroprotective roles of flavonoid “hispidulin” in the central nervous system: A review

**DOI:** 10.22038/IJBMS.2024.76605.16573

**Published:** 2024

**Authors:** Saeed Mustapha, Rabiu Abdussalam Magaji, Mohammed Garba Magaji, Ibrahim Bako Gaya, Baraka Umar, Yusuf Yusha’u, Abubakar Bishir Daku, Samaila Musa Chiroma, Aliyu Jaafar, Mohamad Zulfadli Mehat, Che Norma Mat Taib, Mohamad Aris Moh’d Moklas

**Affiliations:** 1 Department of Human Physiology, Faculty of Basic Medical Sciences, Federal University Dutse, PMB 7156, Jigawa State, Nigeria; 2 Department of Human Physiology, Faculty of Basic Medical Sciences, Ahmadu Bello University (ABU), 810107, Zaria, Nigeria; 3 Department of Pharmacology and Therapeutics, Faculty of Pharmaceutical Sciences, Ahmadu Bello University (ABU), 810107, Zaria, Nigeria; 4 School of Health Sciences, Universiti Sains Malaysia, Kuban Kerian, Kelantan, Malaysia; 5 Neucastle University Medicine Malaysia (NuMed), No. 1, Jalan Serjana 1, Kota ilmu, 79200 Iskandar Puteri (formerly Nusajaya) Johor-Malaysia; 6 Department of Human Anatomy, Faculty of Basic Medical Sciences, Ahmadu Bello University (ABU), 810107, Zaria, Nigeria; 7 Department of Anatomy, Faculty of Medicine and Health Science, University Putra Malaysia (UPM), Serdang 43400, Selangor, Malaysia

**Keywords:** Brain. CNS, Flavone, Inflammation, Oxidative stress

## Abstract

Interest in naturally occurring phytochemicals has been on the increase, they are believed to reduce the risk of brain disorders. Hispidulin (HN) is a phenolic flavonoid compound with various pharmacological and biological effects on the central nervous system. It belongs to the flavone class of flavonoids. It can be found in different plant materials, especially fruits and vegetables. The literature used in this review was collected from credible scientific databases including ScienceDirect, Scopus, PubMed, Google Scholar, and Hindawi without time restriction, using relevant keywords, such as HN, brain, central nervous system, flavonoids, and flavones. HN was discovered to possess pro-apoptotic properties, act as an antioxidant, inhibit cytokine production and toll-like receptor 4 expression, as well as impede nuclear factor kappa beta and mitogen-activated protein kinase B. HN was also found to inhibit lipid peroxidation in vitro and reduce brain edema in mice. These pharmacological potentials suggest that HN is a promising candidate for neuroprotection in CNS disorders like depression and epilepsy. This review provides an update on the scientific literature concerning how these activities could help provide various forms of neuroprotection in the CNS. Additional experimental data on the effects of HN in models of neurological disorders and neuroprotection should be explored further. Based on the current study, HN is a promising candidate for neuroprotection of the CNS.

## Introduction

Hispidulin (HN) is a flavone class of flavonoids that occurs naturally with several biological activities, flavonoids are polyphenols derived from plant products (1-3). It is a flavonoid found in traditional medicines (4). It has the chemical name 4′, 5, 7-trihydroxy-6-methoxyﬂavone, which can be found in different vegetables and fruits. Some studies have demonstrated its role as an antifungal, antioxidant, anti-thrombotic, antineoplastic, and anti-osteoporotic agent (5, 40). Additional research has demonstrated that the compound HN exhibits a wide range of biological functions such as anti-inflammatory, anti-cancer (suppression of cancer cells), and antioxidant effects. It has also been shown to ameliorate osteoporosis and drug-induced hepatotoxicity (hepatic protection) in rodents (3, 6-10). It was also demonstrated that HN shows low toxicity in both *in vitro* and *in vivo* experimental models (11). According to Dai *et al*. (9) HN can promote apoptosis and inhibit invasion and migration of nasopharyngeal carcinoma cells (CNE-2Z) *in vitro* and prevent tumor growth *in vivo* by regulating PI3K/AKT (phosphatidylinositol-3 OH kinase/ serine/threonine protein kinase) mechanisms (11). The administration of HN has also been shown to suppress cardiac hypertrophy, cerebral ischemia-reperfusion injury, and skin inflammation (12, 13) and to penetrate the blood-brain barrier (BBB)(2). It possesses antiepileptic activity and contributes to the reduction of glutamate release by attenuating voltage-dependent calcium entry and by directly interacting with the exocytotic machinery (2). In a study, a xenograft mouse model was used to test the *in vivo *therapeutic activities of HN at doses of 40 mg/kg and 20 mg/kg, both doses significantly suppressed tumor growth (7). Treatment with HN significantly reduced kainic acid-induced hippocampal neuronal cell death. This protective effect is accompanied by the suppression of microglial activation and subsequent production of proinflammatory cytokines such as interleukin-6, interleukin-1β, and tumor necrosis factor-alpha in the hippocampus (14). 

Flavonoids constitute a class of natural phenolic compounds present in vegetables, fruits, and herbal medicine (15). They provide color and flavor to fruits and vegetables and include six major subgroups: flavanones, flavanols, flavones, isoflavones, and anthocyanidins. Flavonoids also exist in plants as glucoside derivatives, with O-glycosidic bonds formed with carbohydrates such as D-glucuronic acid, D-glucose, and D-galactose (16). Studies have shown that more than 6000 flavonoid types have been isolated from natural sources (17). Moreover, flavonoids have a low molecular weight and fifteen carbon skeletons. They also contain two benzene rings linked by a heterocyclic pyran ring (18). The recommended daily dietary intake is 50 to 800 mg (19). Flavones are the most extensively studied flavonoids due to their promising properties in some *in vivo* and *in vitro* studies** (**20). The presence of a double bond between the C2 and C3 skeleton and a ketone at position 4 of their ring distinguishes flavones (e.g., HN) from other flavonoid types (21). C-glycoside flavones showed greater stability and resistance to acidic, enzymatic, and alkaline hydrolysis compared to O-glycoside flavones, hence their diverse activities (22). The biological effects of flavones on many health conditions are associated with their antioxidant, anti-inflammatory, antiviral, and anti-carcinogenic activities. These processes are mediated by their interplay with different key enzymes and signaling processes involving transcription factors and cytokines (23). Apigenin is the most explored natural bioactive flavone owing to its potential therapeutic functions** (**24). Other neuroprotective flavones include vitexin, baicalein (25), HN, luteolin, luteoloside, baicalin, chrysin, diosmin, nobiletin, scutellarin, ginkgetin, orientin, and tricin (26). Preclinical, clinical, and epidemiological studies have investigated the neuroprotective effects of natural compounds using *in vitro* and *in vivo* models (26).

However, updated data on the effects of HN in neuroprotection remain to be explored (27). The increasing need for nutraceuticals has led to greater demand for new sources of bioactive substances. Thus, there is increasing interest in investigating plants with potential pharmacological importance (10). This review aims to provide up-to-date scientific literature concerning how the biological and pharmacological activities of HN could help provide various forms of neuroprotection and explore its potential for treating various CNS conditions.

***Chemical properties of hispidulin***

**Figure 1:**
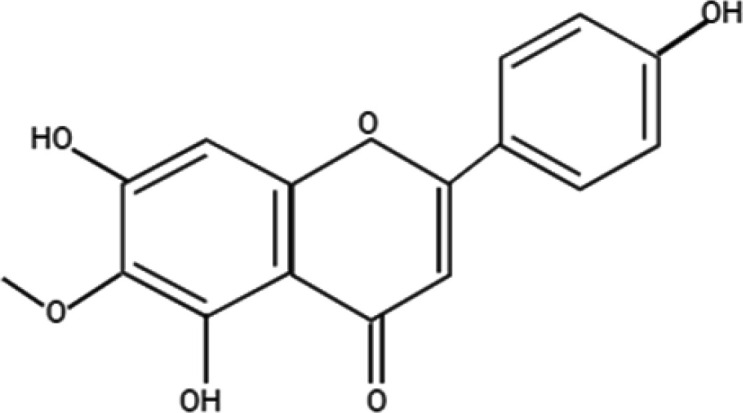
Chemical structure of Hispidulin (HN)

HN has a chemical structure of 4′,5,7-trihydroxy-6-methoxyﬂavone (Figure 1). It is a potent benzodiazepine (BZD) receptor ligand with positive allosteric properties. It is a monomethoxyflavone, scutellarein methylated at position 6 (scutellarein 6-methyl ether). HN is also called dinatin and has the molecular formula C_16_H_12_O_6_ (28) with a molecular weight of 300.26. HN can be quantified with High-Performance Liquid Chromatography (HPLC) and High-Performance Thin-Layer Chromatography (HPTLC) (29) techniques. According to a previous study, flavonoids effectively decrease the concentration of key intermediates by directly reacting with phenylacetaldehyde to form the corresponding adducts (30). Kavvadias *et al*. (31) reports that this HN probably acts as a partial positive allosteric modulator at *γ*-Aminobutyric acid (GABA_A_) receptors and penetrates the blood-brain barrier (BBB). 

***Safety profile of hispidulin***

HN possesses more than 95% purity along with other phytoconstituents as determined by HPLC when separated from the Phyla nodiflora* (P. nodiflora)* sample in a one-step separation (32). Moreover, HN showed low toxicity in nasopharyngeal carcinoma cells (CNE-2Z) cancer-bearing mice at a dose of 20 mg/kg/day (9). During an *in vivo* toxicity assessment of *Anvillea radiata* aqueous extract performed on Wistar rats, HN was identified as one of the phytocompounds exhibiting the strongest binding affinity for the main protease (M^pro^) target of SARS-CoV-2 (33) with no signs of toxicity. *Tamarix ramosissima* bark extract, isorhamnetin, HN, and cirsimaritin significantly inhibited the formation of 2-amino-1-methyl-6-phenylimidazo [4, 5-b] pyridine, which is the most abundant heterocyclic amine in foods, with the highest inhibitory rate of 72.92% (30).

***Sources and bioavailability of HN***

HN is commonly found in fruit skin, buckwheat, red pepper, red wine, and tomato skin, and is regularly consumed in daily foods (34). It occurs in Artemisa and Salvia species which are widely accepted as traditional medicinal plants (35). Medicinal plants with high contents in HN include Asteraceae *Centaurea cyanus, Centaurea malacitana, Centaurea hierapolitana, Aegialophila pumila, Centaurea pichleri, Centaurea phyllocephala, Centaurea phyllocephala, Grindelia Argentina, Centaurea clementei, Centaurea furfuracea,* *Centaurea malacitana *and also in several *Artemisia* and *Salvia* species (36). Flavones such as HN have a robust chemical diversity and are widely present in higher plants as glycosides or aglycones (35). The low bioavailability of flavonoids can be attributed to their low permeability to the intestinal membrane. After oral administration, HN exhibited rapid plasma concentration, and the isomer pair of diosmetin and HN did not show any significant differences in the pharmacokinetic profile (37). The study of Kim & Lee (4) discovered that HN undergoes rapid absorption in the stomach and intestines, resulting in an absolute bioavailability of 4.02% following oral administration. Moreover, HN has also been documented to exhibit effective penetration for topical delivery owing to its physicochemical and structural characteristics, achieving percutaneous absorption within 3 hr (38).

***Pharmacological Roles of HN***

*Role of HN in neurotoxicity and apoptosis*

The protective activities of HN against neurotoxicity have been demonstrated (6). In a study, pretreatment with HN decreased infarct area, decreased brain edema, and improved brain function in rats (8). Moreover, HN significantly attenuated kainic acid-induced hippocampal neuronal cell death, and this protective effect was followed by suppression of microglial activation and production of proinflammatory cytokines such as interleukin-1β, interleukin-6, and tumor necrosis factor-α in the rat hippocampus (14). In another study, bupivacaine-challenged mouse neuroblastoma N2a cells were treated with HN, and neuronal injury was assessed by examining cell apoptosis and viability. These results indicate that treatment with HN substantially attenuated bupivacaine-induced cell injury. There was also an increase in the levels of phospho-AMPK and phospho-GSK3β (AMP-activated protein kinase/glycogen synthase kinase 3β) and suppression of bupivacaine-induced loss of mitochondrial membrane potential. Another current research showed that HN suppressed allergic inflammatory reactions by decreasing histamine release and inflammatory cytokines such as interleukin-4 (IL-4) and tumor necrosis factor-α (TNF-α) in cells (39)(Table 1). Apoptosis is a key pattern of neuronal death and plays a major role in pathogenesis and prognosis. It is also caused by the activation of cysteine-aspartic proteases (Caspases), which regulate cell death, hydrolyze, and cleave relevant proteins in aged rats (40). In a previous study, the pan caspase-3 inhibitor FMK attenuated the apoptotic effects of HN. These findings imply that HN-induced apoptosis and cell death are mediated through a caspase-dependent mechanism (41). Another recent study showed that astrocytes can adopt neurotoxic phenotypes that improve disease progression. Astrocytes are multitasking cells that contribute to central nervous system defense, homeostasis, and immunity (42). HN administration was also found to promote temozolomide-induced apoptosis according to Chen *et al.* (43). Their results suggested that a combination of HN and temozolomide could improve the antitumor effects of temozolomide against malignant gliomas. Moreover, an experimental result showed that HN improved cell viability and slowed down apoptosis in LPS-treated BV2 microglia cells (44). Furthermore, it can be deduced that HN protects against neurotoxicity or cell injury, but enhances apoptosis in cancer models** (**antitumor effects)**. **According to Woo *et al*. (45), HN enhances sensitivity for anti-cancer drugs, such as temozolomide, sunitinib, and tumor necrosis factor-related apoptosis-inducing ligand (TRAIL). It does not increase apoptosis alone, but increases sensitivity to these drugs, resulting in induction of apoptosis.

*Antiepileptic property of HN*

**Figure 2:**
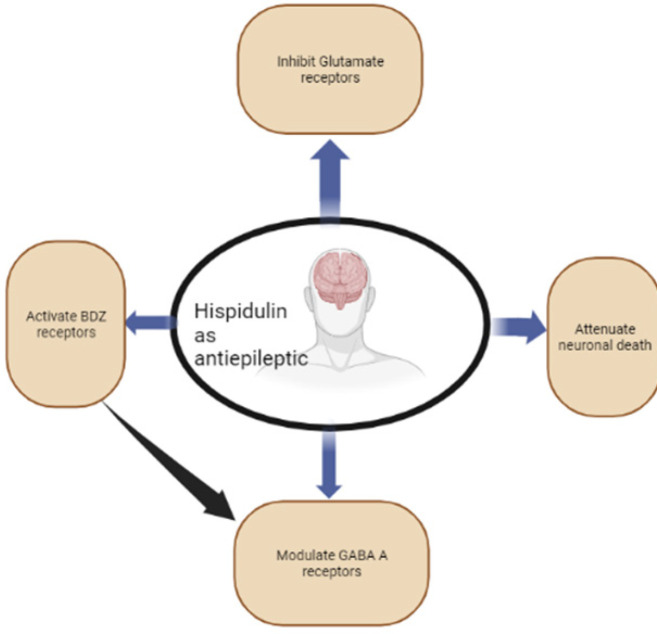
Schematic diagram showing the antiepileptic effects of hispidulin in CNS

HN exhibits antiepileptic properties (46). HN was **and is** capable of passing through the blood-brain barrier (BBB) and exerting anticonvulsant antiepileptic effects (2). BBB serves as a strict control point that regulates the influx of several compounds from the periphery into the brain and is detected in all its regions, with the exception of the circumventricular organs. It plays a fundamental role in neuronal function (47). Excessive release of glutamate (an excitatory neurotransmitter) is believed to be related to the neuropathology of epilepsy (48). HN inhibits the release of glutamate caused by the K⁺ channel blocker 4-aminopyridine (4-AP) in cortical synaptosomes through the suppression of presynaptic voltage-dependent Ca^2+^ entry and the extracellular signal-regulated kinase 1 and 2 (ERK/synapsin I) signaling pathway in rats (49). An *in vitro* study has also shown that HN can activate modulators of human benzodiazepine (BZD) receptors and interact with binding sites on the GABA A receptor complex that inhibits CNS functions (40)(Figure 2). The results of a study (14) indicated that the flavone HN exerts anticonvulsive activity and suppresses the death of neuronal cells in animal models of kainic acid-induced seizures. This finding enhances our understanding of the actions of HN in the brain. It suggests that this natural substance is valuable for treating epilepsy and other brain disorders related to neuroinflammation and excitotoxicity. In a Seizure-prone, male Mongolian gerbil epilepsy model, HN and diazepam (10 mg/kg and 2 mg/kg per day, respectively) decreased the number of animals with seizures after seven days of administration (30 and 25%, respectively compared with 80% in the vehicle group)(31). HN from sage (*Salvia officinalis*) served as an anticonvulsant that acts as a positive allosteric modulator on recombinant α1β2γ2 GABA A receptors that are partially blocked by flumazenil (50). Furthermore, HN demonstrated a flumazenil-sensitive anticonvulsant action similar to diazepam, in seizure-prone Mongolian gerbils used as animal models of epilepsy (51).

*Effects of HN in stroke and ischemia/reperfusion*

**Figure 3:**
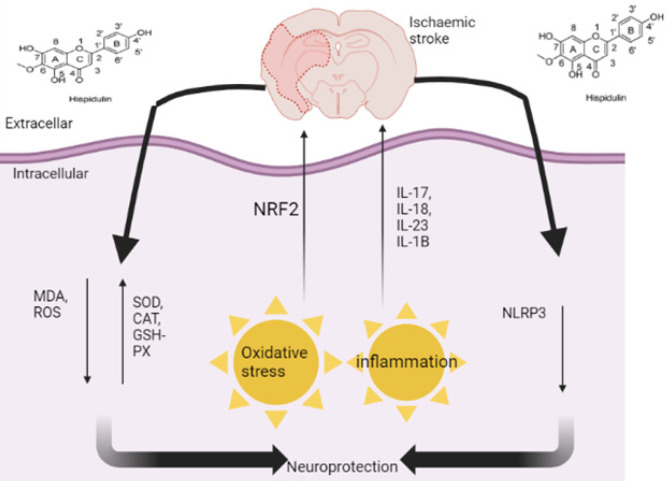
Schematic diagram showing the effects of HN on ischemia and reperfusion

Stroke occurs when the blood supply to brain tissue decreases or stops (16). It is categorized as either ischemic or hemorrhagic, with ischemic strokes comprising over 80% of all cases (52). Emerging studies have demonstrated that polyphenols, such as HN, have antioxidant potential, and play a neuroprotective role after ischemic stroke. In a Wistar rat model, HN injected 50 mg/kg daily for 7 days intraperitoneally, has shown up-regulation of superoxide dismutase (SOD), glutathione peroxidase (GSH-Px), catalase (CAT), and nuclear factor erythroid 2- related factor 2 (NRF2), and down-regulation of malondialdehyde (MDA) and reactive oxygen species (ROS)(53)(Figure 3). Ischemia or reperfusion injury is a major cause of neurological deficits following stroke (8). Oxidative stress after stroke is considered the key factor in brain injury caused by cerebral ischemia. Excessive ROS production in the human body initiates oxidative stress caused by stroke (54). An *in vivo* and *in vitro* study showed that HN exerted a protective effect against ischemia and reperfusion injury by inducing the NRF2 antioxidant pathway through modulation of AMP-activated protein kinase or glycogen synthase kinase 3β (AMPK/GSK3β) signaling (55). In another study by An *et al.* (8), HN has shown a positive role in the neurological symptoms of rats after ischemia or reperfusion injury, in addition to reducing infarct size and brain edema. Moreover, HN exerted its neuroprotective effects *in vivo *and *in vitro *by suppressing NOD-LRR- and pyrin domain-containing protein 3 (NLRP3) mediated pyroptosis and inducing the NRF2 antioxidant pathway. These results suggest that HN may be an important neuroprotective agent against ischemia or reperfusion injury.

*Pro-cognitive effects of HN*

Cognition refers to mental processes associated with knowledge acquisition (56). It entails processes associated with memory, judgment, attention, reasoning, evaluation, problem-solving, and decision-making (56). Flavonoids have direct functions in memory acquisition, consolidation, and storage. Their effects on the vascular system may induce an increase in cerebral blood which has an impact on acute cognition, or may lead to an increase in hippocampal vascularization capable of inducing more neuronal growth. For example, fisetin (10 mg/kg), a flavanol found in strawberries, has also been shown to improve long-term potentiation and enhance recognition of objects in male mice (26). A previous study (6) provided evidence of neuroprotective effects of HN against sevoflurane-induced memory dysfunction. Both Y-maze and novel object recognition tests showed that memory dysfunction was significantly attenuated by pretreatment with HN in aged rats, and also revealed that HN reversed sevoflurane-induced amyloid beta (Aβ) accumulation (Table 1) and neuroinflammation. The cognitive properties of HN from Cirsium rivulare suggest that it possesses pro-cognitive activity (57). 

*Effects of HN on anxiety and depression*

Jia *et al* . (58) suggested that flavonoids may improve the symptoms of depression and anxiety. Flavonoids such as rutin, hesperidin, and quercetin have been reported to have antidepressant-like effects (59-61). Repeated flavonoid administration increased the time spent in the aversive-like context but did not affect the locomotor activity and the number of steps climbing in the staircase test in mice (59). HN from *C. rivulare* has been studied for its anxiolytic properties, and results suggested that it possesses anxiolytic effects (45). HN inhibits methamphetamine-induced hyperlocomotion at IP doses without affecting apomorphine-induced hyperlocomotion (62). It was also found that intracerebellar microinjection of HN, which is an active constituent of the *C. inerme *ethanolic extract, decreased methamphetamine-induced hyperlocomotion, but neither induced sedation nor impaired motor tone in mice through activation of alpha 6-containing GABA_A_ receptors (63). Moreover, HN also has significant BDZ-like activities, including motor impairment and anxiety. HN isolated from the medicinal plant, *Artemisia herba-alba*, was found to be an antagonist or weak partial agonist of the BDZ binding site and inhibited the binding of diazepam to membranes of the rat brain *in vitro* by mixed competitive and non-competitive mechanisms (64). Hipsidulin (1 mg/kg, 3 mg/kg, 10 mg/kg, and 30 mg/kg, IP) isolated from *Salvia triloba* exerted significant antidepressant-like effects in the tail suspension and forced swimming tests. It also significantly increased the brain γ-aminobutyric acid levels and decreased brain glutamate levels after the forced swimming test. These outcomes suggest the involvement of glutamatergic and GABAergic mechanisms in the antidepressant-like effects of HN (60).

*Antioxidant activity of HN*

**Figure 4:**
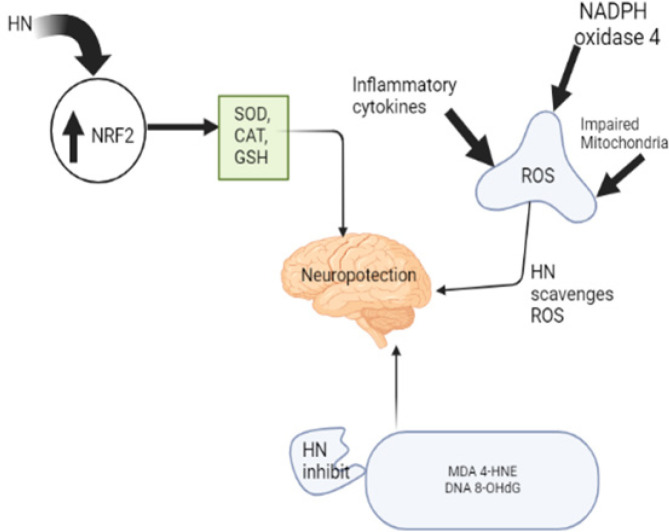
Schematic diagram showing the effects of HN on oxidative stress

Oxidative stress is an intracellular imbalance between ROS and the antioxidants that participate in defense against ROS (65). HN shows high antioxidant potential and inhibits lipid peroxidation *in vitro* (14). Another finding has associated the antioxidant properties of HN to be predominantly by activating NRF2 which serves as a master regulator of the cellular antioxidant defense against environmental insults in the body (66). Dysfunctional mitochondrial activity leads to the buildup of ROS caused by leakage from the impaired electron transport chain. HN has been verified to enhance energy metabolism and suppress oxidative stress (13). Lipid oxidation due to 4-hydroxynonenal (4-HNE) and malondialdehyde, and also oxidation of DNA due to 8-hydroxy-2-deoxyguanosine (8-OHdG) were also inhibited by the administration of HN (Figure 4)(67-69). These compounds have been utilized for evaluating oxidative stress in animal models across different diseases, traumatic brain injury for example. The antioxidant effect of HN was linked to the down-regulation of nicotinamide adenine dinucleotide phosphate hydrogen (NADPH**)** oxidase 4, activation of catalase, superoxide dismutase activities, and the restoration of glutathione concentrations (Table 1)(3). HN was evaluated for the scavenging activity of free radicals and tyrosinase inhibitory effect in cell-free systems. However, it showed high antioxidant potential at all tested concentrations (70). In addition, HN isolated from Indian medicinal plants was tested for its activity as an inhibitor of microsomal lipid peroxidation and free radical scavenging of oxygen *in vitro*, and as a xenobiotic model of toxicity in mice. HN inhibits lipid peroxidation *in vitro* and further administration of HN to mice after bromobenzene intoxication reduces serum glutamate-pyruvate transaminase activity (48). According to Kut *et al*. (71), there are conflicting findings on the antioxidant activities of HN. The reactivity of HN in the ferric ion reducing antioxidant power (FRAP) assay and 2, 2-Diphenyl-1-picrylhydrazyl (DPPH) reduction assay was low (0.09 and 0.019 of the reactivity of Trolox). The results showed the necessity of caution in translating the antioxidant effects assayed in simple cell-free systems for more complex systems.

*Anti-inflammatory role of HN*

A lot of plant derivatives have been used as anti-inflammatory agents (72). Of the several flavones, HN has shown the strongest binding affinity (37). In addition, some evidence has proposed that inflammation may contribute to the symptoms of various psychiatric disorders (73). The anti-inflammatory effects of HN have been reported recently by Kim *et al*. (3, 74). They showed that HN administration attenuates different inflammatory diseases in animal models by reducing cytokine production and TLR_4_ expression, inhibiting NFK_B_ and MAPK cascades, and alleviating immune cell infiltration (Table 1). HN also prevents the adhesion of monocytes to the vascular endothelium and inhibits NF-қB, MAPKs, and AKT signaling in endothelial cells (75). Studies have shown that inflammatory cytokines are produced not only by immune cells but also by CNS cells in the body, including neurons in response to insults (47). Cytokines are low-molecular-weight glycoproteins or regulatory proteins secreted by various cells in the body, including white blood cells that participate in chronic and acute inflammation via interactions with the complex network (76). Most cytokines are referred to as interleukins (ILs), a name depicting that they are secreted by some leukocytes and act on other similar cell types. Two other important types of cytokines are interferons (IFNs), which are capable of activating immune cells such as macrophages, natural killer (NK) cells, and tumor necrosis factors (TNFs), which are known to cause cell death (47). Cytokines like TNF-𝛼 and IL-6 are proinflammatory (responsible for initiating, propagating, and maintaining the inflammatory process)(77). HN inhibits the release of IL-1β, IL-8, TNF-α, IL-6, and intercepts activation of NF-kB signaling in uric acid nephropathy rats according to Lin *et al.* (75). The inflammatory response is primarily driven by pro-inflammatory cytokines produced by T cells, macrophages, and natural killer cells in response to immune system activation (78). Kang *et al*. (46) explored the anti-inflammatory activity of HN with a focus on the skin. HN was administered orally (0.1, 1, and 10 mg/kg) for five consecutive days for a duration of three weeks. For the *in vitro* study, keratinocytes were pretreated with HN (0.1, 1, and 10 μM) and then stimulated with TNF-α and interferon (IFN)-γ. Oral administration of HN reduced keratinization, cell infiltration, redness, ear thickness, and immunoglobulin IgE and IgG2a levels in a dose-dependent manner. In keratinocytes, HN pre-treatment suppressed the expression of TNF-α/IFN-γ-induced cytokines such as interleukin IL-6, IL-8, IL-1β, and chemokines.

**Table 1:** Summary of the neuroprotective effects of hispidulin on the central nervous system (CNS)

Role of hispidulin	Processes/Mechanisms	References
Neurotoxicity and apoptosis	Decrease brain edema, decrease hippocampal neuronal cell death, Suppress IL-4, IL-6, IL-1B, TNFα, and histamine release, induce apoptosis	(14, 8, 9, 41)
Antiepileptic	Activate benzodiazepine (BDZ) receptors, inhibit glutamate release, attenuate neuronal death, anticonvulsant, and modulate GABA_A _receptors	(6, 50, 51)
Stroke and ischemia	Decrease brain edema, suppress NLRP_3 _receptors, induce NRF_2 _pathway, up-regulate NRF_2_, SOD, GSH-Px, and CAT, down-regulate MDA and ROS	(8, 54, 56)
Pro-cognitive	Reversed memory dysfunction by attenuation of Aβ accumulation and neuroinflammation	(40, 58)
Anxiety and depression	Increase GABA, decrease glutamate, inhibit hyperlocomotion, and antagonize BDZ binding sites	(61, 63, 65)
Antioxidant	Activate NRF_2, _inhibit lipid peroxidation, inhibit DNA oxidation, down-regulation of NADPH, activation of CAT and SOD, and restoration of glutathione levels	(3, 49, 67)
Anti-inflammatory	Decrease cytokine production, decrease TLR_4 _expression, inhibit NFK_B_ and MAPK	(3, 48, 74)

Hispidulin activates the BZD receptor and interacts with the binding sites on the GABA A receptor complex, which inhibits the CNS. Hispidulin inhibits the release of glutamate (an excitatory neurotransmitter) in the CNS and attenuates neuronal death associated with epilepsy

BZD: benzodiazepine; GABA A: Gamma aminobutyric acid A; CNS: Central nervous system

HN induced up-regulation of SOD, CAT, and GSH-Px, and down-regulation of MDA and ROS due to oxidative stress via NRF2. It reduces neuroinflammation caused by IL-17, 18, 23, and 1B through suppression of NLRP3. These pathways provide neuroprotection to the brain during ischemia

HN, hispidulin; NRF2, nuclear factor erythroid 2- related factor 2; NLRP3, NOD- LRR- and pyrin domain-containing protein 3; IL, Interleukin; SOD, superoxide dismutase; GSH-PX, Glutathione peroxidase; CAT, catalase; MDA, malondialdehyde; ROS, reactive oxygen species

HN act on NRF2 to regulate SOD, CAT, and GSH to prevent oxidative stress. It also inhibits MDA 4-HNE and DNA 8-OHdG. HN scavenges ROS generated from impaired mitochondria, cytokines, and NADPH oxidase 4

HN, hispidulin; NRF2, nuclear factor erythroid 2-related factor 2; SOD, superoxide dismutase; GSH, Glutathione peroxidase; MDA 4-HNE, malondialdehyde 4-hydroxynonenal; DNA 8-OHdG, 8-hydroxy-2-deoxyguanosine; ROS, reactive oxygen species; NADPH oxidase 4, Nicotinamide adenine dinucleotide phosphate hydrogen oxidase 4

## Conclusion

The major pharmacological and beneficial properties of HN in this review include antioxidant, anti-inflammatory, antidepressant, pro-cognitive, and antiepileptic activities, and HN serves as an apoptosis inducer. Most of these properties provide neuroprotection to the CNS. Based on the current study, HN is a promising neuroprotective candidate. Further studies on this natural product, and determining its mechanism of action along with new drug design and delivery techniques will provide an alternative to conventional neuroprotective drugs.
